# PanelDesign: Integrating Epidemiological Information into the Design of Diagnostic NGS Gene Panels

**DOI:** 10.3390/genes13040684

**Published:** 2022-04-13

**Authors:** Jörg Schmidtke, Peter Philipp, Kathrin Rommel, Ralf Glaubitz, Jörg T. Epplen, Michael Krawczak

**Affiliations:** 1 amedes MVZ wagnerstibbe, Georgstrasse 50, D-30159 Hannover, Germany; ralf.glaubitz@amedes-group.com (R.G.); joerg.epplen@amedes-group.com (J.T.E.); 2 Medizinische Hochschule Hannover, Carl-Neuberg-Strasse 1, D-30625 Hannover, Germany; 3 amedes Medizinische Dienstleistungen GmbH, Haferweg 40, D-22769 Hamburg, Germany; peter.philipp@amedes-group.com; 4 Bundesinstitut für Arzneimittel und Medizinprodukte (BfArM), Kurt-Georg-Kiesinger-Allee 3, D-53175 Bonn, Germany; kathrin.rommel@bfarm.de; 5 Institute of Medical Informatics and Statistics, Kiel University, University Hospital Schleswig-Holstein Campus Kiel, Brunswiker Strasse 10, D-24105 Kiel, Germany; krawczak@medinfo.uni-kiel.de

**Keywords:** genomics, England, panelapp, orphadata, eurogentest/ESHG, recommendations, core genes, genetic epidemiology

## Abstract

We report upon PanelDesign, a framework to support the design of diagnostic next generation DNA sequencing panels with epidemiological information. Two publicly available resources, namely Genomics England PanelApp and Orphadata, were combined into a single data set to allow genes in a given NGS panel to be ranked according to the frequency of the associated diseases, thereby highlighting potential core genes as defined by the Eurogenetest/ESHG guidelines for diagnostic next generation DNA sequencing. In addition, PanelDesign can be used to evaluate the contribution of different genes to a given disease following ACMG (American College of Medical Genetics) technical standards.

## 1. Introduction

Next generation DNA sequencing (NGS) technologies have replaced Sanger sequencing in many routine settings of genetic diagnostics for rare diseases. This holds particularly true for conditions with a high level of locus heterogeneity, including many cardiovascular diseases, autism as well as intellectual disability, and for diagnostic situations with little clinical specificity such as, for example, ‘severe pediatric disorders’ or ‘fetal anomalies’. This notwithstanding, current NGS implementations targeting predefined sets of genes (‘panels’), partial or entire exomes, or complete genomes, often entail substantially higher false positive and false negative rates than Sanger sequencing, mainly due to insufficient sequencing coverage [1,2].

In view of these shortcomings, current Eurogentest/ESHG guidelines for diagnostic NGS [3] recommend that “only genes with a […] confirmed […] relationship between the aberrant genotype and the pathology” should be tested, and that any transition from Sanger sequencing to NGS must not eclipse the proven analytical sensitivity of the former for most, if not all, so-called ‘core gene’ sets. Core genes are loosely defined as “genes that are responsible for a significant proportion of the defects” (ibid) with which they are associated. In order “to avoid irresponsible testing, for the benefit of patients, ‘core disease gene lists’ should be established by the clinical and laboratory experts” (ibid).

So far, the above plea to the scientific community has been answered only for a relatively small number of diseases, mostly heritable cardiovascular conditions and familial cancer. For the great majority of diseases potentially amenable to NGS diagnostics, core genes still remain undefined and the expected level of diagnostic accuracy (labeled Type A, B, or C test, as proposed by Matthijs et al. [3]) is unclear. A recently published American College of Medical Genetics and Genomics (ACMG) technical standard does not even refer to a core gene concept at all, but instead demands Type A sequencing accuracy for “mutational hotspots and sites of common founder variants” and allows “regions with minor contribution to disease or genes added to the panel as part of a broader differential diagnosis” to be excluded from the test [4]. Moreover, if known, designing a gene panel must of course take any molecular characteristics of the candidate genes into account that could impede the diagnostic accuracy of the panel, including an enrichment with deletions or duplications, or the presence of large homologous regions or pseudogenes. In the worst case, such genes would have to be excluded as well. In any case, it appears worth mentioning in this context that lack of diligence in terms of the composition and performance of diagnostic gene panels may also have medico-legal consequences [5].

An outstanding facilitator of sharing, accessing, and evaluating gene panels for routine genetic diagnostic testing is the Genomics England PanelApp [6]. PanelApp is an expert-curated tool that integrates information on gene-disease associations taken from databases such as ClinGen, with other relevant resources, including the Human Phenotype Ontology (HPO). As of August 2021, PanelApp included 308 panels of diverse clinical specificity, ranging from a single gene (e.g., CHARGE syndrome) via several hundred genes (e.g., ‘adult onset movement disorder’ or ‘autism’) to well over 1000 (e.g., ‘fetal anomalies’) or even several thousand genes (e.g., ‘severe paediatric disorder’). Within each panel, genes are labeled either green, amber, or red, signifying ‘high’, ‘moderate’, or ‘not enough’ evidence, respectively, for an association with the condition in question. However, no attempt has been made so far to rank green genes according to the proportion of cases caused in the sense of the Eurogentest/ESHG guideline.

Other resources, such as GeneReviews [7], refer to detection rates and thereby provide indirect guidance to the definition of core genes, but as yet no sensible rate cut-offs have been proposed to this end. Furthermore, GeneReviews are typically specific for a single phenotype or a clinically narrow group of conditions. They are therefore not very helpful for ranking genes according to their relative importance in broader disease groups.

One major problem of the Eurogentest/ESHG definition of core genes is that it may not necessarily apply in each and every context because the etiological relevance of a gene may vary according to the ethnicity, age, or other characteristics of the probands. While core genes should therefore ultimately be personalized, taking epidemiological information into account in the definition process would appear to be a first step in the right direction. A useful resource in this regard is the Orphadata Rare Diseases Epidemiology Dataset [8], which includes various types of prevalence figures from different geographic regions alongside the respective disease names and Orphacodes of the diseases, disease groups, or disease subtypes in question. To the best of our knowledge, this is the only publicly available epidemiological register of rare diseases.

In the following, we describe PanelDesign, a framework to support the design of NGS gene panels with epidemiological information. It combines the aforementioned public resources into a single data set that allows genes in a panel to be ranked according to the frequency of the associated diseases, thereby highlighting potential core genes as defined by the Eurogenetest/ESHG guidelines for diagnostic NGS [3].

## 2. Materials and Methods

**Figure 1:**
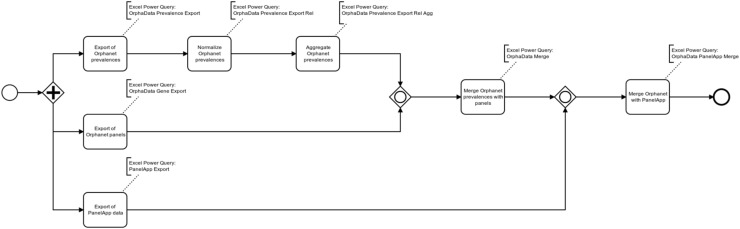
The multistep process of integration of the Orphadata and PanelApp resources into PanelDesign.

The integration of the Orphadata and PanelApp resources into PanelDesign followed a multistep process (Figure 1).

First, the entire PanelApp panel collection of genes was extracted (source PanelApp API) and elements irrelevant to the purpose of core gene definition were removed from the original JSON structures (step ‘Export of PanelApp data’, Figure 1).

Second, the ‘genes associated with rare diseases’ section of Orphadata (product6) [9] was exported and irrelevant elements were deleted from the original XML structures (step ‘Export of Orphanet panels’, Figure 1). All use of Orphadata material complied with creative commons rules [10]. In the following, Orphadata field names [11] are given in italics for better recognition.

Third, the ‘rare disease epidemiology’ file was extracted from Orphadata (product9) [12] and cleared of irrelevant elements as well. Orphadata contains both relative and absolute, literature-based disease frequencies. Absolute frequencies are provided as numbers of (independent) cases or families with a given disease reported from a geographic region, whereas relative frequencies are given as prevalence per 100,000 inhabitants. For better comparability, both frequencies were normalised to the same scale as follows: If the *PrevalenceType* field in Orphadata contained the phrase ‘Cases/families’ (i.e., if absolute case numbers were reported), then the PrevalenceValMoyRel variable defined in PanelDesign was set to the absolute frequency × 100,000 divided by the population size of the region (as determined through an internet search). Otherwise, PrevalenceValMoyRel was set to the original prevalence per 100,000 inhabitants from Orphadata (steps ‘Export Orphadata prevalences’, ‘Normalize Orphadata prevalences’, Figure 1).

Next, a filter was applied to confine the extraction of prevalence figures to the following geographical regions relevant to patients of central European descent: Austria, Belgium, Canada, Czech Republic, Denmark, Europe, France, Germany, Ireland, Italy, Luxembourg, The Netherlands, North America, Poland, Portugal, Spain, Sweden, Switzerland, United Kingdom, and United States. In addition, we also extracted data labelled ‘worldwide’ in the Orphadata field *PrevalenceGeopraphic*. Two additional filters were applied to confine the extraction process to prevalence figures that were larger than zero and that were labelled ‘validated’ in the Orphadata field *Status*.

The normalized Orphanet prevalence figures were then aggregated by grouping them according to a combination of *DisorderOrphaID*, *DisorderName* and a suitable aggregation of *PrevalenceType* (i.e., distinguishing between prevalence and incidence figures only), and by calculating the following four PanelDesign metrices (step ‘Aggregate Orphanet prevalences’, Figure 1):

MinPrevalenceValMoyRel: minimum PrevalenceValMoyRel per group
MaxPrevalenceValMoyRel: maximum PrevalenceValMoyRel per group
MidPrevalenceValMoyRel: mean PrevalenceValMoyRel per group
MedPrevalenceValMoyRel: median PrevalenceValMoyRel per group

The datasets resulting from steps ‘Export Orphanet panels’ and ‘Aggregate Orphanet prevalences’ were next joined by their *DisorderOrphaID*. This step (‘Merge Orphanet prevalences with panels’, Figure 1) yielded a combination of aggregated disease prevalence figures and disease-associated genes.

Finally, the datasets resulting from steps ‘Export PanelApp data’ and ‘Merge Orphanet prevalences with panels’ were linked by the corresponding gene symbol (step ‘Merge Orphanet data with PanelApp’, Figure 1). For ease of practical use, the combined datasets were finally sorted by PanelApp panel ID (level 1, descending), aggregated Orphadata *PrevalenceType* (level 2, descending) and PanelDesign variable MidPrevalenceValMoyRel (level 3, descending).

## 3. Results

The core result making up the essential part of PanelDesign is an Excel sheet, called ‘PanelApp_Merge’, in which the two Orphadata resources are combined with the complete PanelApp gene list. This Excel sheet is part of a file that also contains the PanelApp and Orphadata resource files alongside relevant meta data, such as the population sizes used. The file is accessible as an online Supplementary Material accompanying the present report.

Almost all PanelApp panels (307 of 308) contained genes associated with diseases of known prevalence. High specificity is attained for the definition of core genes when locus heterogeneity is low and the prevalence of the associated disease is high. If a gene-disease association is unequivocal, any gene associated with the most prevalent condition within a group of diseases targeted by a given panel is identified as a core gene. It should be noted that testing genes exclusively associated with the most frequent condition within a group of diseases, targeted by a given panel, does not necessarily provide the highest relative diagnostic yield.

## 4. Discussion

The utility of PanelDesign as a means of identifying potential core (or contributing) genes for NGS-based diagnostics is crucially dependent upon the granularity of the gene-disease associations provided by the underlying disease classification system. Thus, Orphadata prevalence figures are related to the diseases associated with the respective gene via the Orphacode of the condition. In the Orphanet disease classification system [13], an Orphacode may relate to a group of disorders, or a defined disorder, or a subtype of a disorder. Whenever mutations in a given gene can be lined to more than one clinical entity, as defined by Orphacodes, PanelDesign will rank this gene in a panel according to the frequency of the gene-associated diseases, not the relative contribution of mutations in that gene to disease causation.

Many disease subtypes are defined on the basis of disease-gene associations, but the original disease itself may not always be linked, in the epidemiological resource file, to the genes defining its subtypes. Marfan syndrome (Orphacode 558), for example, is a disease with two genetically defined subtypes, namely Marfan syndrome type 1 (Orphacode 284963) and Marfan syndrome type 2 (Orphacode 284973). Epidemiological data are however only available for Orphacode 558, but not for Orphacodes 284963 and 284973, so that the two subtypes do not appear in PanelDesign.

Whenever an Orphacode relates to a disease that is associated with mutations in a gene that is not associated with any other condition, then the disease frequency is a direct measure of the relative diagnostic importance of the gene. For example, ‘childhood onset dystonia or chorea’ (PanelApp panel 847) is most frequently due to propionic acidemia, which in turn is caused exclusively by mutations in the *PCCA* and *PCCB* genes. Therefore, the two genes are clearly identified as core gene candidates for a panel targeting childhood onset dystonia or chorea.

When using PanelDesign in its present form, the ranking, in a panel, of genes with multiple disease associations must be manually assessed for its plausibility with a view to the targeted phenotypic spectrum. For example, for nephrolithiasis (PanelApp panel 149), *SLC9A3*, *SLC4A*1, and *SLC6A19* appear on the top of the gene list. This is, however, due to the fact that *SLC9A3* is also a cystic fibrosis modifier gene, *SLC4A1* is also associated with spherocytosis, and *SLC6A19* also causes Hartnup disease, all of which are comparatively frequent conditions. In the nephrolithiasis panel, *AGXT* should instead be considered the primary core gene candidate. Moreover, *SLC4A1* and *SLC6A19* are part of the panel because they are associated with distal renal tubular acidosis (Orphacode 93608), and iminoglycinuria (Orphacode 42062), respectively, two co-morbidities of nephrolithiasis. The ranking of the genes in the panel does not, however, depend upon this etiological link because no epidemiological data are available in Orphadata for Orphacodes 93608 and 43062. Such need for scrutiny may seem to be a disadvantage, but it could sometimes have the beneficial side effect of alerting the PanelDesign user to differential diagnoses not hitherto considered (in the sense of ‘reverse phenotyping’). This notwithstanding, if ever so desired, the ambiguities just described could be resolved easily by assigning each gene-disease association a unique Orphacode.

In PanelDesign, relative disease frequencies (mainly birth prevalence or incidence, and point prevalence) were directly adapted from Orphadata, whereas absolute frequencies (number of cases or families) were converted to relative frequencies reminiscent of a period prevalence. All three frequency categories potentially could be useful in a more personalized approach to defining core genes. For example, birth prevalence could be the category of choice if the patient to be diagnosed is still very young. Ethnicity could be another criterion for the personalization of core genes. In the version of PanelDesign reported here, we used epidemiological information acquired in regions enriched with populations of central European descent. Information from other populations is still extremely scarce. However, with more such data being systematically collected and incorporated, sets of ethnicity-specific core genes could be developed.

## 5. Conclusions

PanelDesign is a framework to support the design of diagnostic next generation DNA sequencing panels with epidemiological information. Two publicly available resources, Genomics England PanelApp and Orphadata, were combined into a single dataset that allows genes to be ranked according to the frequency of the associated diseases. At this stage, the practical applicability of PanelDesign is still somewhat limited owing to a lack of appropriate epidemiological data. However, our report provides a proof of principle of how to use the latter to inform the design of diagnostic NGS panels, and we call upon the scientific community to improve the data situation in this regard. Necessary efforts would include both enhancing the granularity of the disease nomenclature, in the sense of increasing the specificity of gene-disease associations, and putting more emphasis upon the collection of genetic epidemiological data, especially from non-European populations.

## Data Availability

Not applicable.

## Supplementary Materials

The following supporting information can be downloaded at: https://www.mdpi.com/article/10.3390/genes13040684/s1. Supplementary Materials is provided as an excel sheet containing the following data sets: Master Data. Statistics; PanelApp Merge; OrphaData Prevalence Rel; OrphaData Prevalence Rel Agg; Orphadata Gene; Orphadata Merge; Orphadata Regions; PanelApp Export; OrphaData Prevalence Agg.
